# Serotonin–norepinephrine reuptake inhibitor antidepressant effects on regional connectivity of the thalamus in persistent depressive disorder: evidence from two randomized, double-blind, placebo-controlled clinical trials

**DOI:** 10.1093/braincomms/fcac100

**Published:** 2022-04-15

**Authors:** Jie Yang, David J. Hellerstein, Ying Chen, Patrick J. McGrath, Jonathan W. Stewart, Bradley S. Peterson, Zhishun Wang

**Affiliations:** Department of Psychiatry, and National Clinical Research Center for Mental Disorders, The Second Xiangya Hospital of Central South University, Changsha 410011, Hunan, China; Department of Depression Evaluation Service, New York State Psychiatric Institute, 1051 Riverside Drive, Unit #51, New York, NY 10032, USA; Department of Depression Evaluation Service, New York State Psychiatric Institute, 1051 Riverside Drive, Unit #51, New York, NY 10032, USA; Vagelos College of Physicians and Surgeons, Columbia University, New York, NY 10032, USA; Department of Depression Evaluation Service, New York State Psychiatric Institute, 1051 Riverside Drive, Unit #51, New York, NY 10032, USA; Vagelos College of Physicians and Surgeons, Columbia University, New York, NY 10032, USA; Mailman School of Public Health, Columbia University, New York, NY 10032, USA; Department of Depression Evaluation Service, New York State Psychiatric Institute, 1051 Riverside Drive, Unit #51, New York, NY 10032, USA; Vagelos College of Physicians and Surgeons, Columbia University, New York, NY 10032, USA; Department of Depression Evaluation Service, New York State Psychiatric Institute, 1051 Riverside Drive, Unit #51, New York, NY 10032, USA; Vagelos College of Physicians and Surgeons, Columbia University, New York, NY 10032, USA; Institute for the Developing Mind, Children’s Hospital Los Angeles, Los Angeles, CA 90027, USA; Keck School of Medicine, University of Southern California, Los Angeles, CA 90089-9021, USA; Department of Depression Evaluation Service, New York State Psychiatric Institute, 1051 Riverside Drive, Unit #51, New York, NY 10032, USA; Vagelos College of Physicians and Surgeons, Columbia University, New York, NY 10032, USA

**Keywords:** persistent depressive disorder, functional connectome, graph theory, serotonin noradrenaline reuptake inhibitor antidepressant, placebo

## Abstract

Previous neuroimaging studies have shown that serotonin–norepinephrine reuptake inhibitor antidepressants alter functional activity in large expanses of brain regions. However, it is not clear how these regions are systemically organized on a connectome level with specific topological properties, which may be crucial to revealing neural mechanisms underlying serotonin–norepinephrine reuptake inhibitor treatment of persistent depressive disorder. To investigate the effect of serotonin–norepinephrine reuptake inhibitor antidepressants on brain functional connectome reconfiguration in persistent depressive disorder and whether this reconfiguration promotes the improvement of clinical symptoms, we combined resting-state functional magnetic resonance imaging (fMRI) scans acquired in two randomized, double-blind, placebo-controlled trial studies of serotonin–norepinephrine reuptake inhibitor antidepressant treatment of patients with persistent depressive disorder. One was a randomized, double-blind, placebo-controlled trial of 10-week duloxetine medication treatment, which included 17 patients in duloxetine group and 17 patients in placebo group (ClinicalTrials.gov Identifier: NCT00360724); the other one was a randomized, double-blind, placebo-controlled trial of 12-week desvenlafaxine medication treatment, which included 16 patients in desvenlafaxine group and 15 patients in placebo group (ClinicalTrials.gov Identifier: NCT01537068). The 24-item Hamilton Depression Rating Scale was used to measure clinical symptoms, and graph theory was employed to examine serotonin–norepinephrine reuptake inhibitor antidepressant treatment effects on the topological properties of whole-brain functional connectome of patients with persistent depressive disorder. We adopted a hierarchical strategy to examine the topological property changes caused by serotonin–norepinephrine reuptake inhibitor antidepressant treatment, calculated their small-worldness, global integration, local segregation and nodal clustering coefficient in turn. Linear regression analysis was used to test associations of treatment, graph properties changes and clinical symptom response. Symptom scores were more significantly reduced after antidepressant than placebo administration (*η*^2^ = 0.18). There was a treatment-by-time effect that optimized the functional connectome in a small-world manner, with increased global integration and increased nodal clustering coefficient in the bilateral thalamus (left thalamus *η*^2^ = 0.21; right thalamus *η*^2^ = 0.23). The nodal clustering coefficient increment of the right thalamus (ratio = 29.86; 95% confidence interval, −4.007 to −0.207) partially mediated the relationship between treatment and symptom improvement, and symptom improvement partially mediated (ratio = 21.21; 95% confidence interval, 0.0243–0.444) the relationship between treatment and nodal clustering coefficient increments of the right thalamus. Our study may indicate a putative mutually reinforcing association between nodal clustering coefficient increment of the right thalamus and symptom improvement from serotonin–norepinephrine reuptake inhibitor antidepressant treatments with duloxetine or desvenlafaxine.

## Teaser

Yang *et al*. reported that serotonin–norepinephrine reuptake inhibitor antidepressants have an effect on the regional connectivity of the thalamus in patients with persistent depressive disorders, and there is a putative mutually reinforcing association between regional connectivity increments of the right thalamus and symptom improvement from serotonin–norepinephrine reuptake inhibitor antidepressant treatments.

## Graphical Abstract

**Graphical Abstract:**
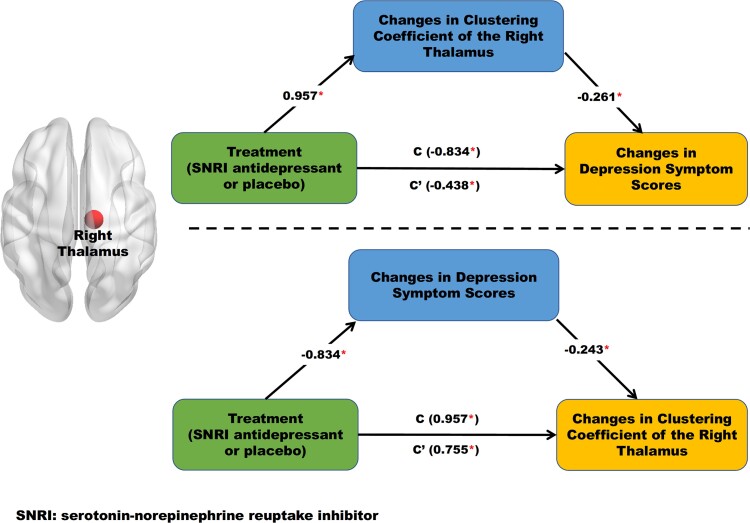

## Introduction

Non-major chronic depression is common, with a prevalence as high as 1.5–5% in the general population.^1^ It can lead to significant functional impairments, high rates of health care utilization, increased unemployment and use of public entitlements.^2^ Because of its chronicity, by definition lasting at least two years, non-major chronic depression results in greater psychosocial burden, more functional impairments, and greater suicidality than episodic major depressive disorder (MDD).^3,4^ The DSM-5^5^ recognized the importance of chronicity by consolidating various forms of chronic depression including dysthymia disorder (DD), residual major depression, and coexisting major depression and dysthymia, into a single category of persistent depressive disorder (PDD).

In terms of antidepressants used in current clinical practice, the dual-action serotonin noradrenaline reuptake inhibitors (SNRIs) are among the first-line agents. As with MDD, although clinical symptoms of PDD often respond to SNRI antidepressant treatment, up to half of depressed patients either do not respond or have side-effects leading to premature discontinuation of treatment.^6^ Understanding the causal mechanisms of the antidepressant response, especially the response to first-line agents, can help to develop more effective treatments.

In recent years, longitudinal neuroimaging research has demonstrated that SNRI antidepressants can alter brain networks of depressive patients, including reduction of hyperconnectivity in the default-mode network^7^ and in the thalamo-cortico-periaqueductal circuit,^8^ as well as regulating functional connectivity of the cortico-striatal circuit.^9^ SNRI antidepressants appear to alter functional activity in large expanses of brain regions, but most researchers have limited their examinations to focal brain activity. Furthermore, the approach of quantifying functional connectivity of a network/circuit by averaging connectivity strength is agnostic to network structure and ignores the interconnecting pattern of these nodes (i.e. brain connectome). Depicting topological properties of the brain network and linking them to certain morphological mechanisms that underpin psychiatric disorders has become a prevailing analytic method in mental health research.^10,11^

The human brain is constructed in a small-world manner, with a highly clustered/segregated neighbourhood of brain regions and occasional integrative long-distance connections for conferring high efficiency of information processing at relatively low connection cost.^12,13^ Among major depressive patients, previous studies have consistently identified a sub-optimal small-world topology organization across multiple modalities.^14–17^ It has been reported that antidepressant medications can optimize a sub-optimal functional connectome to a more small-world pattern^18^ by regulating the strength of short- or long-distance functional connectivity.^19^ Our prior study also indicated that SNRI antidepressants can enhance the regional segregation of the morphology covariance network in patients with PDD.^20^ Taken together, we extend our prior investigations by investigating how SNRI antidepressants affect the topological organization of functional connectome in the resting-state, and how the altered topological properties may bring about clinical symptom improvement in PDD.

The current study aimed to examine how functional connectome topology is affected by SNRI antidepressants in patients with PDD in two randomized double-blind, placebo-controlled trials (RCTs). The RCT study design enabled us to ascertain whether SNRI antidepressants cause topological changes of the brain functional connectome in patients with PDD, rather than merely inferring topological changes induced by medication. We used a hierarchical strategy to examine the topological properties changes affected by SNRI antidepressants. To this end, small-worldness, normalized clustering coefficient (a measure of segregation), normalized characterized path length (a measure of integration), and nodal clustering coefficient were calculated in turn; small-worldness is a measure of the balance between segregation and integration.^12,13^ Considering our previous finding of the strengthening effect of SNRI antidepressants on clustering coefficient,^20^ we expected that SNRI antidepressant treatment would promote a global reconfiguration of the functional connectome, especially its local segregation. The clustering coefficient of each node was further calculated to locate specific brain areas that carry the local segregation changes. We hypothesized that areas with nodal measure changes would be located in the regions observed in previous SNRI antidepressant studies.^8,9^ We also employed linear regression analysis to probe the relationship between treatment, topological property changes, and depressive symptom improvement.

## Materials and methods

### Participants

We combined resting-state fMRI scans from two placebo-controlled RCTs of SNRI antidepressant medications in patients with PDD because the sample size of each study alone was too small, with insufficient statistical power to detect changes caused by antidepressants. One was an RCT of 10-week duloxetine medication (Data set 1) treatment conducted between 26 January 2007 and 22 November 2011, and the other was an RCT of 12-week desvenlafaxine medication (Data set 2) treatment conducted between 5 August 2012 and 28 January 2016.

Data set 1 and Data set 2, respectively, comprised 65 and 59 adults diagnosed with PDD. All were free of significant medical problems. Inclusion criteria allowed enrolment of males and females aged between 20 and 65 years; who scored > = 12 on the 24-item Hamilton Depression Rating Scale (HAMD)^21,22^ at baseline; who had a current Diagnostic and Statistical Manual of Mental Disorders, Fourth Edition (DSM-IV) diagnosis of DD or depression NOS; and who were deemed likely to be compliant with study procedures. Exclusion criteria included DSM-IV diagnosis of major depression in the past 3 months; bipolar disorder; schizophrenia or other psychotic disorders; dementia or other cognitive impairment; drug or alcohol abuse or dependence within the past 6 months; current psychoactive medication use (≥2-week washout of antidepressants was required); serious risk for suicide during the course of the study; unstable medical conditions; current or planned pregnancy; current eating disorder; and lack of capacity to consent to study participation. Of 65 subjects in Data set 1 enrolled in the study, 34 received fMRIs at baseline and week 10, and of 59 subjects in Data set 2 enrolled in the study, 33 received fMRIs at baseline and week 12 (see Table 1). The consort flow diagram is presented in Supplementary Fig. 1.

**Table 1:** Demographic and clinical characteristics of 65 patients with PDD

	Antidepressant group (*n* = 33)	Placebo group (*n* = 32)	Statistics
Age (years)	38.3 ± 12.43	36.78 ± 10.63	*P* = 0.6^a^; *t* = 0.53
Sex (M/F)	11/22	19/13	*P* = 0.035*^a^; χ^2^ = 4.43
Race or ethnicity			
White	20	22	*P* = 0.7^a^; χ^2^ = 2.17
African American	5	6	
Hispanic	4	1	
Asian	3	2	
Not specified	1	1	
HAMD scores			
Baseline	20.27 ± 4.96	20.38 ± 4.35	*P* ^b^ < 0.001*, *F* = 13.52; (a2 versus a1) *P* < 0.001*; (b2 versus b1) *P* < 0.001*; (a1 versus b1) *P* = 0.93;
Follow-up	7.06 ± 4.8	13.44 ± 8.2	
First-onset age (years)	18.64 ± 12.14	15.88 ± 8.12	*P* ^a^ = 0.29; *t* = 1.08
WDOI (years)	18.45 ± 14.41	18.69 ± 14.62	*P* ^a^ = 0.95; *t* = −0.66
DOCE (month)	130.67 ± 122	116.47 ± 118.14	*P* ^a^ = 0.64; *t* = 0.48
Anxiety			
Current	14	11	N/A
Past	11	12	N/A
Prior substance abuse	6	8	N/A
Recurrent	9	10	N/A
Mean FD			
Baseline	0.1 ± 0.05	0.09 ± 0.06	*P* ^b^ = 0.68, *F* = 0.17; (a2 versus a1) *P* = 0.94 (b2 versus b1) *P* = 0.6; (a1 versus b1) *P* = 0.47;
Follow-up	0.1 ± 0.06	0.09 ± 0.05	

Note: PDD, persistent depressive disorder; *n*, number; HAMD, 24-item Hamilton Depression Rating Scale; WDOI, the whole duration of the illness; DOCE, duration of current episode; a2, follow-up of the antidepressant group; a1, baseline of the antidepressant group; b2, follow-up of the placebo group; b1, baseline of the placebo group; FD, Jenkinsons mean framewise displacement.

^a^ Represents statistics were calculated as (patients who received antidepressant group) versus (patients who received placebo group).

^b^ Represents statistics were calculated as (a2–a1) versus (b2–b1).

Diagnoses were made via clinical interviews by a board-certified research psychiatrist and confirmed with the Structured Clinical Interview for DSM-IV.^23^ Once ascertaining that all inclusion and no exclusion criteria were present, the clinician explained study procedures and obtained agreement from the patient who attested by signing an IRB-approved consent (Duloxetine IRB: 4967/6363R; Desvenlafaxine IRB: 6457).

### Clinical trial

Patients in Data set 1 and patients in Data set 2, respectively, began an RCT of 10-week duloxetine therapy and an RCT of 12-week desvenlafaxine therapy at the Depression Evaluation Service of the New York State Psychiatric Institute after the baseline fMRI session. For the duration of their participation, patients underwent a clinical assessment every 2 weeks with a psychiatrist, and depressive symptoms were serially rated using the HAMD. In dataset 1, duloxetine dosing began at 30 mg daily; in Data set 2, desvenlafaxine dosing began at 25 mg daily. Drug dosing was increased to maximum tolerated according to a fixed schedule (dosing generally being increased by 25 mg of desvenlafaxine or 30 mg of duloxetine every 2 weeks to a maximum of 100 mg of desvenlafaxine or 120 mg of duloxetine). Most subjects were therefore treated with maximal doses (96.5 ± 12 mg of desvenlafaxine or equivalent placebo and 95 ± 27 mg/day of duloxetine or equivalent placebo), limiting the likelihood that symptom severity or duration of illness could impact dosage of study medication.

### Imaging data acquisition and processing

All image data in both data sets were acquired on a GE Signa 3-T whole-body scanner at New York State Psychiatric Institute (*N* = 62) or at the NY Cornell Medical Center MRI Unit (*N* = 5). The two scanning sites used the same type of scanner and the same set of parameters. High-resolution three-dimensional T_1_-weighted scans were acquired with following acquisition parameter: inversion time = 500ms; flip angle = 90^o^; field of view = 25×25 mm; matrix = 256×256; voxel size = 1.0×1.0×1.0 mm^3^. Whole-brain resting-state fMRI data were acquired using a gradient-recalled echo-planar imaging pulse sequence (repetition time = 2200 ms, echo time = 30 ms; flip angle = 90^o^; slice thickness = 3.5 mm; field of view = 24×24 mm; matrix = 256×256; voxel size = 3.75×3.75×3.75 mm^3^ and total volume = 140).

Image data preprocessing was performed using DPABI toolbox.^24^ To adjust for magnetic saturation delay, the first 10 images were discarded and 130 volumes were obtained for preprocessing. The following preprocessing steps were applied: slice timing correction, motion realignment, spatial normalization with the brain template of Montreal Neurologic Institute (MNI), smoothing of full-width at half-maximum = 8 mm. Nuisance covariates including 12 head motion parameters (including derivatives), white matter and CSF signals were regressed out from the blood oxygenation level dependent signals. The global signal was not removed as recent studies have shown illness-related variance in the global signals.^25^ Displaced volumes (framewise displacement > 0.5 mm) were interpolated by nearest-neighbour interpolation.^26,27^ The exclusion criteria for sample selection included the following: (i) head motions larger than a 2.5 mm translation or 2.5° rotation in any direction; (ii) fMRI data failed to normalize to MNI space which is visually inspected by an experienced data analyst.

The preprocessing procedures above were conducted only on those who completed both baseline and follow-up scans—17 on duloxetine and 17 on placebo in Data set 1, and 17 on desvenlafaxine and 16 on placebo in Data set 2. We pooled Data set 1 and Data set 2 for all subsequent analyses, and patients were then divided into antidepressants (34 patients) and placebo group (33 patients). After quality control, a total of 65 patients with PDD (antidepressant group: *n* = 33; placebo group *n* = 32) were included in the final analysis. To present our results more succinctly, we let A denote the antidepressant group and B denote the placebo group; and numbers 1 and 2 denote before and after treatment, respectively. We therefore have the following abbreviations: a1 (baseline of antidepressants), a2 (follow-up of antidepressants), b1 (baseline of placebo), and b2 (follow-up of placebo). No significant differences were found in Jenkinson’s mean framewise displacement (mean FD) of across all groups [treatment-by-time interaction effects *P* = 0.68, *F* = 0.17; antidepressant group (a2 versus a1) *P* = 0.94; placebo group (b2 versus b1) *P = 0.6*; comparison between baselines (a1 versus b1) *P* = 0.47].

### Network construction and properties calculation

The mean time series was extracted from each of the 264 nodes using 5 mm spheres defined by the Power atlas.^28^ A 264 × 264 symmetric matrix was generated for each participant by computing Pearson correlation coefficients between the time series for each pair of ROIs (region of interest). The resultant matrix was converted to normally distributed scores by using Fisher’s *z* transformation, and the variance due to the linear effects of age, gender, and education years was removed to derive the corrected symmetric matrix. Network measures at each density (sparsity) were calculated on the 264×264 weighted adjacency matrices, which were acquired by thresholding the symmetric matrices at a series of network densities, ranging from top 10 to 50% of all connections, with 2% increments, in line with our prior studies.^27,29^ The reason for choosing this density range is that network measures are less prone to non-biological artefacts and noise in this density range.^30^ Negative correlations were set to zero, in line with other studies of functional connectome construction.^31,32^ We did not use binarized matrices as binarization is arbitrary and can result in the loss of important illness-related biological features that can be captured by weighted network approaches.^33,34^ We used the Brain Connectivity Toolbox (http://www.brain-connectivity-toolbox.net) to quantify network measures.

At the global properties level, we calculated sigma (small-worldness) on weighted, undirected networks. Sigma is a ratio of gamma (normalized clustering coefficient refers to the local specialization) to lambda (normalized characterized path length refers to the global integration)^12^ that is,

(1) sigma=gamma/lambda

We also report gamma and lambda values. These normalized topological properties gamma and lambda must be benchmarked against corresponding mean values of null random graphs as following:

(2) $$\mathrm{gamma}=C/C_{\mathrm{null}}$$

(3) $$\mathrm{lambda}=L/L_{\mathrm{null}}$$

where C indicates the clustering coefficient and L indicates the path length. Bullmore *et al.*^12^ has described the calculation details of parameters C and L. We generated 20 null random networks^27,35^ with the same number of nodes, degree, and degree distribution as the network of interest. At the regional properties level, we calculated the nodal clustering coefficient since it strongly relates to the gamma.

### Statistical analysis

Group differences (antidepressant versus placebo) at baseline in demographic, clinical characteristics and behavioural data on 65 patients were analyzed using two-sample *t* test and χ^2^ tests. We then employed the repeated-measure ANOVA method to assess whether treatment differentially altered clinical symptoms and network metrics on the 65 patients across the two treatment arms (i.e. to assess the treatment-by-time interaction), with using the mean FD and prior substance use as covariates. We also assessed treatment effects on clinical symptoms and network metrics separately in the antidepressant- and placebo-treated patients. As network metrics were calculated across densities, we used functional data analysis (FDA)^36^ to synthesize values across densities before conducting statistical analyses. In the FDA, each network metric curve is treated as a function [y = f(x)], and the sum of differences in *y* values is calculated across densities. Furthermore, statistical maps of regional network metrics were generated after multiple comparison analysis with a false discovery rate (FDR) corrected using the Benjamini and Hochberg method with (*P* < 0.05).

### Exploratory analysis

#### Mediation analysis

Linear regression analyses were used to test associations between treatment, network metrics changes and clinical symptom improvement. We defined two types of models. One type of model set the clinical symptoms response as the dependent variable, treatment (SNRI antidepressant medication = 1 and placebo administration = 0) as the predictor, and the change of network metrics as the moderator variable. Another type of model set the change of network metrics as the dependent variable, treatment as the predictor, and clinical symptom response as the moderator variable. Mediation analysis used the PROCESS macro^37^ 3.5 version for SPSS, with a 5000 bias-corrected bootstrap sample for significance testing.

### Data availability

The data that support the findings of this study are available on request from the corresponding author.

## Results

### Demographic characteristics and clinical symptoms

Demographic and clinical characteristics of patients are presented in Table 1. The antidepressant and placebo groups were matched for age, race/ethnicity, first-onset age, current episode duration and lifetime illness duration. Notedly, the proportion of males in the placebo group was larger than that in the antidepressant group. The HAMD score was more significantly reduced after antidepressant than placebo administration (treatment-by-time interaction effects *P* < 0.001, *F* = 13.52, partial eta-squared *η*^2^ = 0.18).

### Network properties

**Figure 1:**
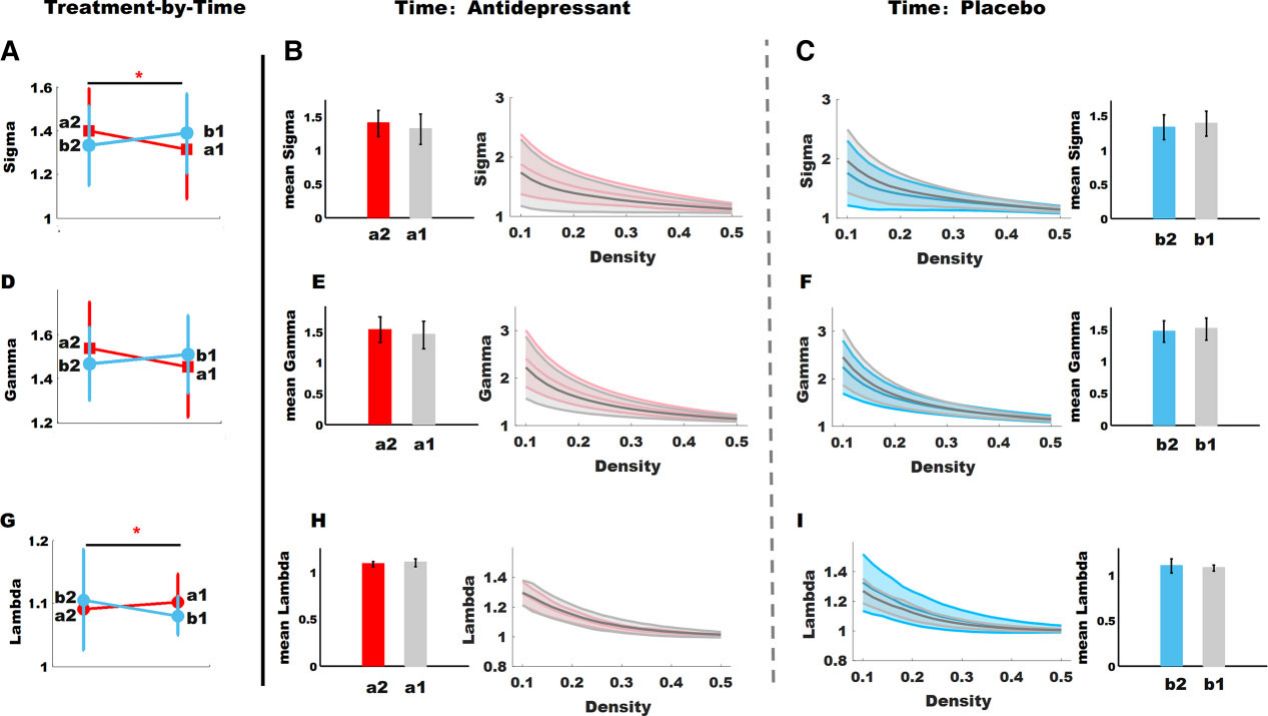
**Longitudinal data analyses to assess changes in global network properties of 65 patients.** (**A**) Sigma showing significant alteration in treatment-by-time interaction (*F* = 4.49, *P* = 0.038); (**B**) comparison of the sigma between follow-up and baseline in the antidepressant group (*P* = 0.09); (**C**) comparison of the sigma between follow-up and baseline in the placebo group (*P* = 0.22); (**D**) Comparison of the Gamma in treatment-by-time interaction (*F* = 3.83, *P* = 0.057); (**E**) comparison of the gamma between follow-up and baseline in the antidepressant group (*P* = 0.09); (**F**) comparison of the gamma between follow-up and baseline in the placebo group (*P* = 0.34). (**G**) Gamma showing significant alteration in treatment-by-time interaction (*F* = 4.16, *P* = 0.044); (**H**) comparison of the gamma between follow-up and baseline in the antidepressant group (*P* = 0.15); (**I**) comparison of the gamma between follow-up and baseline in the placebo group (*P* = 0.14). Symbol ‘*’ represents *P* < 0.05. a2, follow-up of the antidepressant group; a1, baseline of the antidepressant group; b2, follow-up of the placebo group; b1, baseline of the placebo group.

Repeated-measure analysis of variance revealed a significant treatment-by-time interaction effect on sigma (treatment-by-time interaction effects *P* = 0.038, *F* = 4.49, partial eta-squared *η*^2^ = 0.067), and lambda (treatment-by-time interaction effects *P* = 0.046, *F* = 4.16, partial eta-squared *η*^2^ = 0.062). However, it should be noted that there was a significant difference between the lambda of the antidepressant and placebo groups at baseline. Details are presented in Fig. 1 and Table 2.

**Table 2:** Longitudinal data analyses to assess changes in global network properties of 65 patients with PDD

Network properties	Antidepressants group (*n* = 33)		Placebo group (*n* = 32)		ANOVA		Supplementary stats.	
	Follow-up mean (SD)	Baseline mean (SD)	Follow-up mean (SD)	Baseline mean (SD)	*F*	*η*^2^		
Sigma	1.398 (0.2)	1.313 (0.224)	1.331 (0.18)	1.389 (0.18)	4.49*	0.067	(a2 versus a1) *P* = 0.09; (b2 versus b1) *P* = 0.22; (a1 versus b1) *P* = 0.16;	
Gamma	1.537 (0.21)	1.451 (0.223)	1.469 (0.17)	1.51 (0.174)	3.83	0.057	(a2 versus a1) *P* = 0.09; (b2 versus b1) *P* = 0.34; (a1 versus b1) *P* = 0.28;	
Lambda	1.09 (0.026)	1.1 (0.043)	1.11 (0.08)	1.08 (0.03)	4.16*	0.062	(a2 versus a1) *P* = 0.15; (b2 versus b1) *P* = 0.14; (a1 versus b1) *P* = 0.02^a^;	

^a^ Represents *P*<0.05; ***η***^2^ represents partial eta-squared.

a2, follow-up of the antidepressant group; a1, baseline of the antidepressant group; b2, follow-up of the placebo group; b1, baseline of the placebo group.

**Figure 2:**
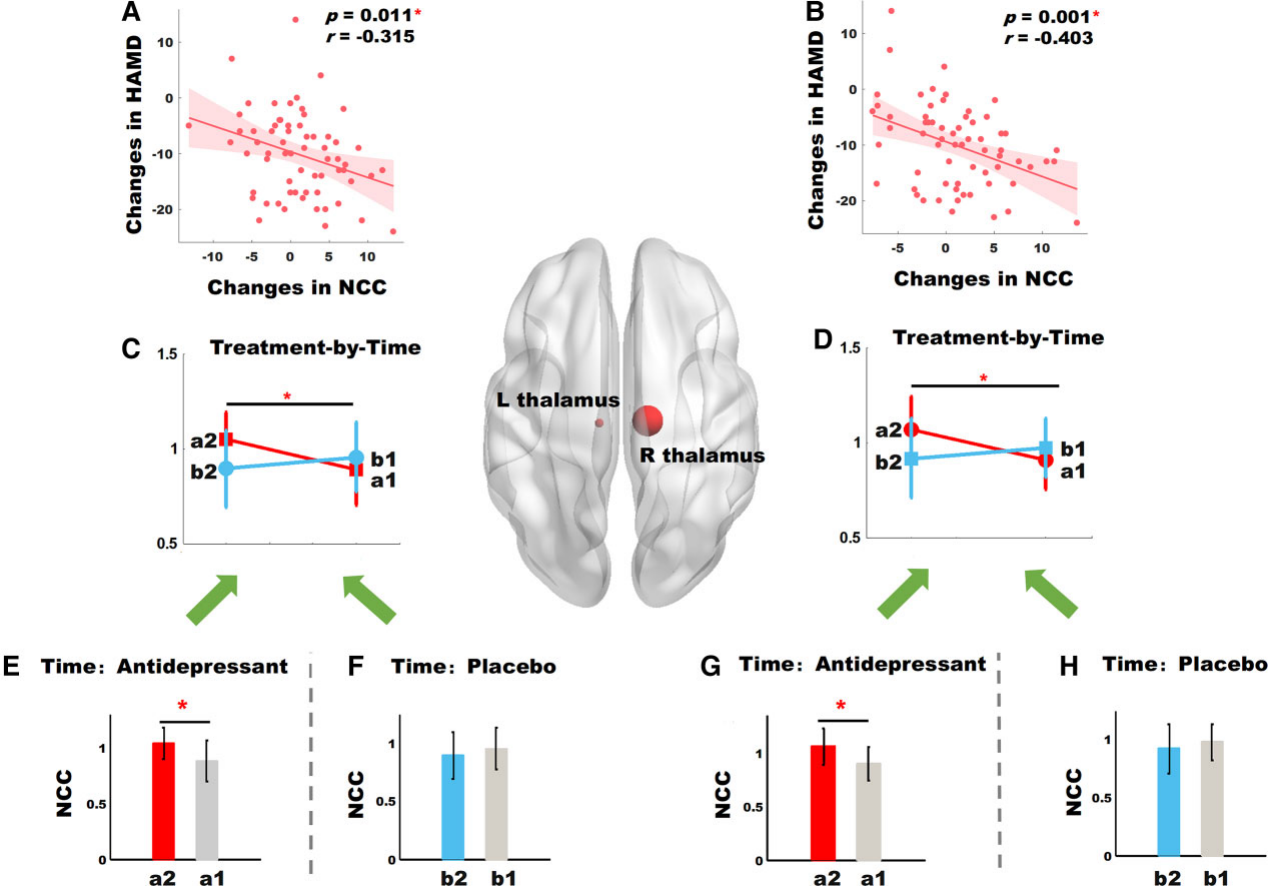
**Longitudinal data analyses to assess changes in regional network properties of 65 patients.** (**A**) The nodal clustering coefficient increment of the left thalamus is positively correlated with the HAMD decrements (*P* = 0.011, *r* = −0.315); (**B**) clustering coefficient of the left thalamus showing significant alteration in treatment-by-time interaction (*F* = 17, *P < 0.001*, *P-corrected* = 0.0144); (**C**) comparison of clustering coefficient of the left thalamus between follow-up and baseline in the antidepressant group (*P < 0.001*); (**D**) comparison of clustering coefficient of the left thalamus between follow-up and baseline in the placebo group (*P = 0.11*); (**E**) The nodal clustering coefficient increment of the right thalamus is positively correlated with the HAMD decrements (*P* = 0.001, *r* = −0.403); (**F**) clustering coefficient of the right thalamus showing significant alteration in treatment-by-time interaction (*F* = 18.8, *P < 0.001*, *P-corrected* = 0.0141); (**G**) comparison of clustering coefficient of the right thalamus between follow-up and baseline in the antidepressant group (*P < 0.001*); (**H**) comparison of clustering coefficient of the right thalamus between follow-up and baseline in the placebo group (*P = 0.12*); Symbol ‘*’ represents *P* < 0.05. a2, follow-up of the antidepressant group; a1, baseline of the antidepressant group; b2, follow-up of the placebo group; b1, baseline of the placebo group; NCC, nodal clustering coefficient; HAMD, 24-item Hamilton Depression Rating Scale.

After further exploring the nodal clustering coefficient (NCC) of 264 nodes, we detected a significant treatment-by-time interaction effect on the left thalamus (treatment-by-time interaction effects *P-corrected* = 0.0144, *F* = 17, partial eta-squared *η*^2^ = 0.21) and the right thalamus (treatment-by-time interaction effects *P*-corrected = 0.0141, *F* = 18.8, partial eta-squared *η*^2^ = 0.23). We also detected that the NCC increment of the bilateral thalamus was positively correlated with symptom decrements (left thalamus *P* = 0.011, *r* = −0.315; right thalamus *P* = 0.001, *r* = −0.403). Details were presented in Fig. 2 and Table 3.

**Table 3:** Longitudinal data analyses to assess changes in regional network properties of 65 patients

Region	MNI	Antidepressants group (*n* = 33)		Placebo group (*n* = 32)		ANOVA		Supplementary stats.
		Follow-up mean (SD)	Baseline mean (SD)	Follow-up mean (SD)	Baseline mean (SD)	*F*	*η*^2^	
L thalamus	(-10–18 7)	1.05 (0.14)	0.89 (0.18)	0.89 (0.2)	0.95 (0.14)	17.0	0.21	(a2 versus a1) *P* < 0.001^a^; (b2 versus b1) *P* = 0.11; (a1 versus b1) *P* = 0.1;
R thalamus	(12–17 8)	1.07 (0.17)	0.91 (0.15)	0.91 (0.21)	0.97 (0.15)	18.8	0.23	(a2 versus a1) *P* < .0.001^a^; (b2 versus b1) *P* = 0.12; (a1 versus b1) *P* = 0.07;

^a^ represents *P* < 0.05; ***η***^2^represents partial eta-squared.

L thalamus, left thalamus; R thalamus, right thalamus; a2, follow-up of the antidepressant group; a1, baseline of the antidepressant group; b2, follow-up of the placebo group; b1, baseline of the placebo group.

### Exploratory analysis

#### Mediation analysis

**Figure 3:**
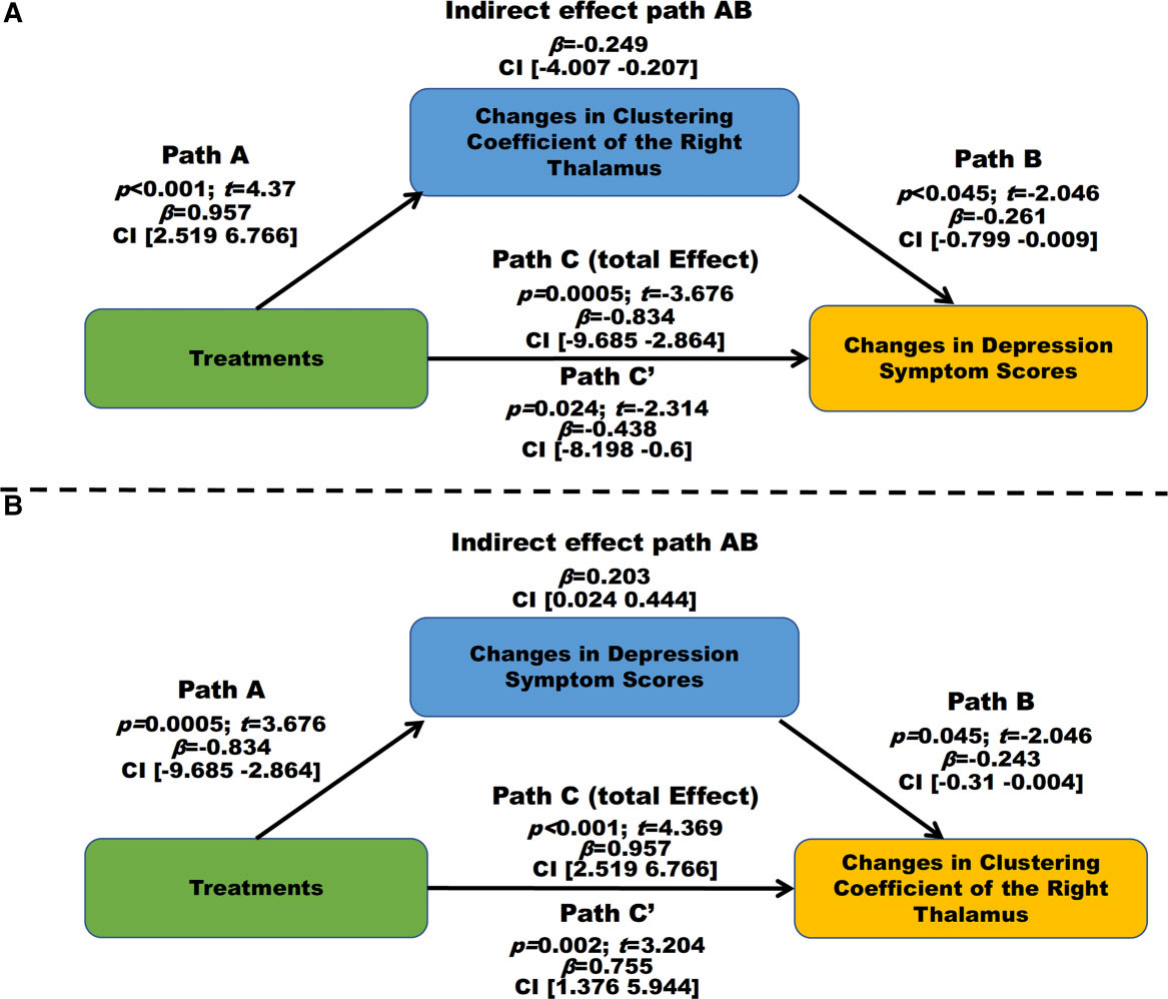
**Longitudinal mediation analyses on 65 patients.** (**A**)The mediation effect of the change of right thalamus nodal clustering coefficient significantly mediated the association between treatment (SNRI antidepressant/placebo) and depressive symptom response. Path C (*t* = 3.68, *P* < 0.001) represents the variance in treatment associated with depressive symptom response, and Path C’ (*t* = 2.314, *P* = 0.024) represents the association between treatment and depressive symptom response after taking into account the change of right thalamus nodal clustering coefficient as a mediator. Path AB (*β* = −0.249, CI [−4.007 −0.207]) is the mediation effect and is significant at *P* < 0.05 based on confidence intervals from bias-corrected bootstrapping of 5000 samples; (**B**)The mediation effect of the HAMD improvement significantly mediated the association between treatment (SNRI antidepressant/placebo) and change of right thalamus nodal clustering coefficient. Path C (*t* = 4.369, *P* < 0.001) represents the variance in treatment associated with the change of right thalamus nodal clustering coefficient, and Path C’ (*t* = 3.204, *P* = 0.002) represents the association between treatment and the change of right thalamus nodal clustering coefficient after taking into account the HAMD improvement as a mediator. Path AB is [*β* = 0.203, CI (0.0243 0.444)] the mediation effect and is significant at *P* < .05 based on confidence intervals from bias-corrected bootstrapping of 5000 samples. SNRI, serotonin noradrenaline reuptake inhibitors; CI, confidence interval.

Given there was a treatment-by-time interaction on increased NCC of the bilateral thalamus and that these increments correlated with clinical symptom improvement, we assessed the association between the bilateral thalamus, treatment and symptom relief. We found that the NCC changes of the right thalamus (effect contribution ratio = −0.249/(−0.249 + −0.438) = 29.86; 95% confidence interval, −4.007 to −0.207) partially mediated the different treatment effects on clinical symptom improvement (details see Fig. 3A), and that symptom improvement [effect contribution ratio = 0.203/(0.203 + 0.755) = 21.21; 95% confidence interval, 0.0243–0.444] partially mediated the different treatment effects on NCC changes of the right thalamus (details see Fig. 3B).

## Discussion

To our knowledge, this is the first longitudinal study to examine changes in topological properties of the functional connectome in placebo-controlled RCTs of SNRI medication treatment of PDD. We report two key observations. First, compared with placebo, SNRI antidepressant medication promotes functional connectome reconfiguration in a more small-world manner; this can be mainly attributable to an increase in global integration. Second, at the regional network metrics level, SNRI antidepressant medication has an effect on the increase of the NCC in the bilateral thalamus, and this increase correlates with decreased symptom scores. Third, longitudinal mediation analyses revealed that NCC changes in the right thalamus partially mediated the relationship between treatment and depressive symptom improvement and that depressive symptom improvement partially mediated the relationship between treatment and NCC changes of the right thalamus.

There was a treatment-by-time interaction effect on small-worldness; this indicated that SNRI antidepressant treatment can promote functional connectome optimization to a more efficient configuration. Compared with HCs, a sub-optimal small-world organization has been consistently observed in depressive patients in terms of the functional connectome in both resting state and task states,^14,15^ as well as the morphological covariance network,^16^ and the diffusion tensor imaging (DTI) tractography-based structural connectome.^15,17^ Consistent with our findings, previous longitudinal studies have highlighted that an aberrant decreased small-worldness of the functional connectome in patients with obsessive-compulsive disorder can be normalized by antidepressant treatment.^18^

How do SNRI antidepressants affect functional connectome to reconfigure in more small-world manner? Our results reveals that this is mainly due to the reduction of characterized path length. The characterized path length of a network is associated with its long-distance connections. Adding some long-distance connections to a randomized network or decreasing the length of long-distance connections of a less small-world network can optimize its organization to higher global integration for more efficient information processing. Consistent with the current study, An *et al.* have reported that antidepressants can increase the connectivity strength of long-distance connections (i.e. an analogy to the reciprocal of the length of long-distance connections) of the functional connectome.^19^ However, it also should be noted that, contrary to our current findings, our prior study^20^ did not detect a treatment-by-time effect on global integration of grey matter covariance. We speculate that this may result from the modality used in our previous study. Morphological covariance has been treated as a surrogate of the structural connectome constructed by DTI tractography^38^ because of apparent system-specific correlation patterns between cortical GM and underlying white matter connectivity.^39^ Future studies using the DTI modality could investigate how SNRI antidepressants affect the structural connectome of patients with PDD. Studies could also investigate the relative timing of alterations in regional clustering and global integration of the functional and structural connectome during SNRI antidepressant treatment. These different approaches could further characterize the mechanism of neural topological reconfigurations caused by SNRI antidepressants.

We also observed that the treatment-by-time interaction has a marginal significant effect on the increase of clustering coefficient. As for strengthen clustering coefficient, previous studies have consistently reported antidepressant effects on enhancing clustering coefficient under resting-state and an emotional regulation task.^18,40^ Our recent study conducted in the same samples reported that SNRI antidepressant treatment strengthened clustering connectivity of the network constructed by grey matter covariance in patients with PDD.^20^ At the regional network metric level, we found there was a treatment-by-time effect on the increased NCC of the bilateral thalamus. The thalamus, a region rich in monoamine neurotransmitters including serotonin and norepinephrine transporters,^41^ has long been confirmed to be one of the target sites of SNRI antidepressants.^8,42^ Davies *et al.*^43^ demonstrated an elevation of regional cerebral blood flow (rCBF) in the thalamus after 6-week venlafaxine treatment. Anand *et al.*^40^ have reported that antidepressants can strengthen regional functional connectivity of the thalamus, amygdala and pallidostriatum during emotional regulation tasks. Notably, a prior study of our group^8^ on the same samples demonstrated an effect of SNRI antidepressants on functional connectivity of the thalamo-cortico-periaqueductal circuit of the pain system. Our current study partially reproduces the findings of our previous investigation by using a graph theory approach from the perspective of network science.

After observing a significant correlation between the NCC change of the bilateral thalamus with clinical symptom relief, we designed two types of linear regression model in longitudinal mediation analyses to further explore the causal relationship between these two factors. Longitudinal mediation analyses revealed that the NCC changes of the right thalamus partially mediated treatment effects on HAMD, and HAMD response partially mediated treatment effect on the NCC change of the right thalamus. These may suggest two types of mechanism, that is, SNRI antidepressants partially alleviate depressive symptoms by increasing the NCC of the right thalamus, and SNRI antidepressants partially increase the NCC of the right thalamus by alleviating depressive symptoms. It seems that the increase of NCC in the right thalamus and the remission of clinical symptoms are mutually reinforcing and that there is no causal relationship between these two factors. These findings suggest that regional connectivity changes of the right thalamus may potentially be used as a reliable parameter to predict remission of clinical symptoms in patients with PDD treated with SNRI antidepressants. Furthermore, they may provide a promising and reliable auxiliary means for the development of new antidepressants. For example, observing the effect of a new antidepressant on regional connectivity of the right thalamus may be an effective approach to predict clinical response.

### Limitations

Our study has several limitations. First, although we pooled samples from two RCT studies, the sample size still remains small, which may induce some statistical Type I error and Type II error. Further investigations with larger samples are needed to replicate our findings. Second, the duration of resting-state data was relatively short (5 min) in an intermediate range of the time needed for stable resting-state data estimates.^44^ Third, our analyses lacked a comparison group of healthy control subjects, so baseline functional connectome properties abnormalities in the patients with PDD could not be determined. Fourth, the number of female subjects in the antidepressant group was significantly more than that in the placebo group. Although we adjusted for gender in our analyses, we cannot entirely exclude its effects as a potential confound. Fifth, most subjects had previous exposure to antidepressants, and thus we cannot exclude the possibility that our findings are attributable to this exposure.

## Conclusion

In summary, we provide the first report of the effects of SNRI antidepressant treatment (duloxetine and desvenlafaxine) within RCTs on the topological metrics of the functional connectome in persistent depressive illness. At the global metric level, SNRI antidepressant medications optimize the functional connectome into a more small-world manner with higher global integration. At the regional metric level, SNRI antidepressants appear to enhance regional connectivity of the thalamus and decrease depressive symptoms, as well as inducing a mutually reinforcing association between regional connectivity of the right thalamus and symptomatic improvement.

## Supplementary Material

fcac100_Supplementary_Data

## Abbreviations

a1 =: baseline of antidepressants
a2 =: follow-up of antidepressants
b1 =: baseline of placebo
b2 =: follow-up of placebo
CI =: confidence interval
DD =: dysthymia disorder
DSM-IV =: Diagnostic and Statistical Manual of Mental Disorders, Fourth Edition
DTI =: diffusion tensor imaging
FD =: framewise displacement
FDA =: functional data analysis
FDR =: false discovery rate
HAMD =: 24-item Hamilton Depression Rating Scale
MDD =: major depressive disorder
MNI =: Montreal Neurologic Institute
NCC =: nodal clustering coefficient
PDD =: persistent depressive disorder
RCTs =: randomized double-blind, placebo-controlled trials
ROIs =: region of interest
SNRIs =: serotonin noradrenaline reuptake inhibitors
