# Supplementary material for: Serotonin–norepinephrine reuptake inhibitor antidepressant effects on regional connectivity of the thalamus in persistent depressive disorder: evidence from two randomized, double-blind, placebo-controlled clinical trials
Source: Brain Commun. 2022 Apr 15;4(3):fcac100. doi: 10.1093/braincomms/fcac100 (PMC9113244; doi:10.1093/braincomms/fcac100)
Supplement: fcac100_Supplementary_Data [file fcac100_supplementary_data.pdf]

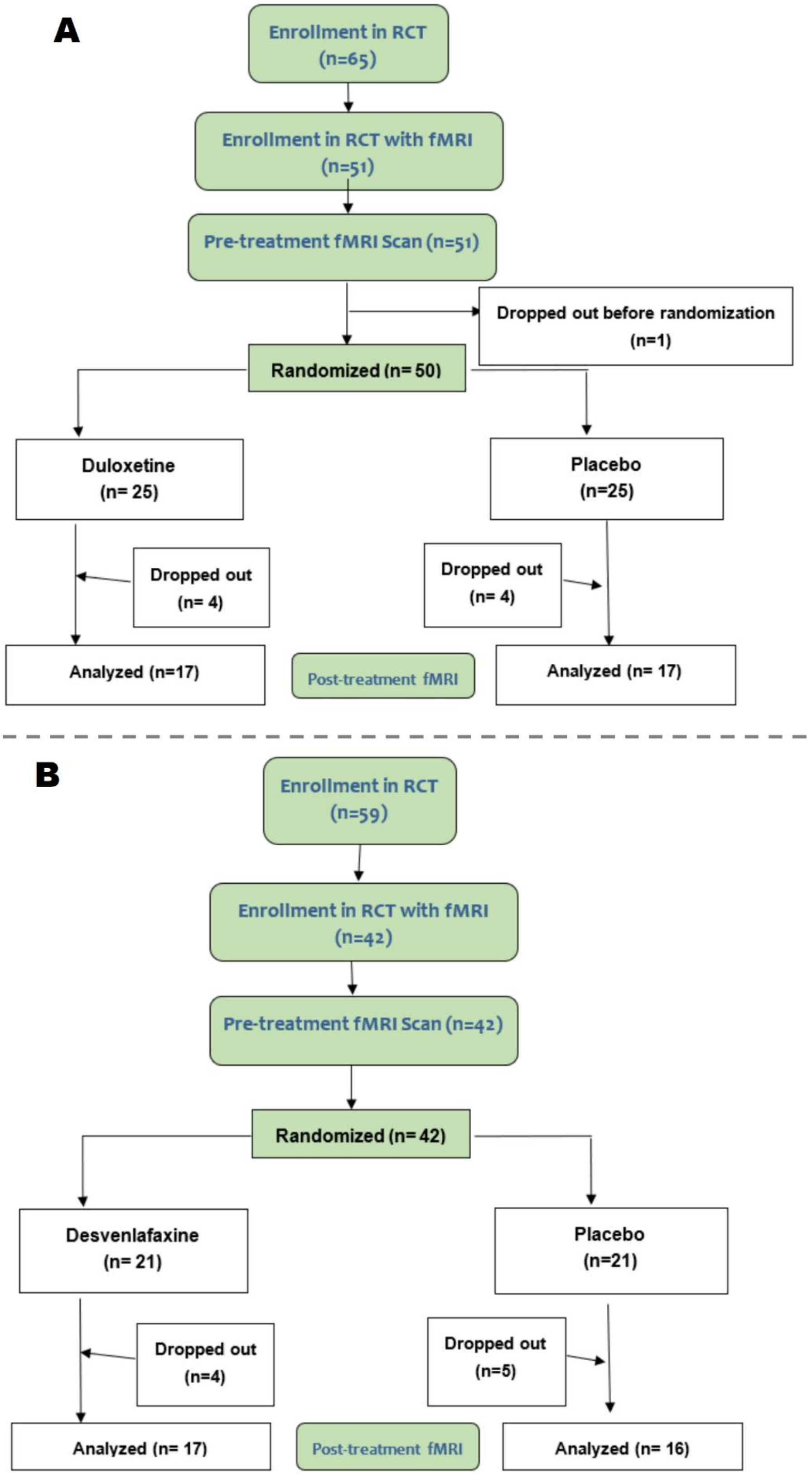

Supplementary Figure 1. Consort flow diagram. (A) Duloxetine study consort diagram; (B) Desvenlafaxine study consort diagram; RCT, randomized double-blind, placebo-controlled trials; fMRI, functional magnetic resonance imaging.
